# *Aspergillus* Species Discrimination Using a Gas Sensor Array

**DOI:** 10.3390/s20144004

**Published:** 2020-07-18

**Authors:** Rosamaria Capuano, Emilia Paba, Antonella Mansi, Anna Maria Marcelloni, Alessandra Chiominto, Anna Rita Proietto, Emiliano Zampetti, Antonella Macagnano, Larisa Lvova, Alexandro Catini, Roberto Paolesse, Giovanna Tranfo, Corrado Di Natale

**Affiliations:** 1 Department of Electronic Engineering, University of Rome Tor Vergata, Via del Politecnico 1, 00133 Rome, Italy; catini@ing.uniroma2.it (A.C.); dinatale@uniroma2.it (C.D.N.); 2 Department of Occupational and Environmental Medicine, Epidemiology and Hygiene, National Institute for Insurance against Accidents at Work (INAIL) Via Fontana Candida 1, 00078 Monte Porzio Catone, Rome, Italy; e.paba@inail.it (E.P.); a.mansi@inail.it (A.M.); a.marcelloni@inail.it (A.M.M.); a.chiominto@inail.it (A.C.); a.proietto@inail.it (A.R.P.); g.tranfo@inail.it (G.T.); 3 Institute of Atmospheric Pollution Research-National Research Council, Via Salaria km. 29300, 00016 Monterotondo, Rome, Italy; e.zampetti@iia.cnr.it (E.Z.); a.macagnano@iia.cnr.it (A.M.); 4 Department of Chemical Science and Technology, University of Rome Tor Vergata, Via della Ricerca Scientifica, 00133 Rome, Italy; larisa.lvova@uniroma2.it (L.L.); roberto.paolesse@uniroma2.it (R.P.)

**Keywords:** *Aspergillus* species, electronic nose, volatile organic compounds, porphyrins

## Abstract

The efficiency of electronic noses in detecting and identifying microorganisms has been proven by several studies. Since volatile compounds change with the growth of colonies, the identification of strains is highly dependent on the growing conditions. In this paper, the effects of growth were investigated with different species of *Aspergillus*, which is one of the most studied microorganisms because of its implications in environmental and food safety. For this purpose, we used an electronic nose previously utilized for volatilome detection applications and based on eight porphyrins-functionalized quartz microbalances. The volatile organic compounds (VOCs) released by cultured fungi were measured at 3, 5, and 10 days after the incubation. The signals from the sensors showed that the pattern of VOCs evolve with time. In particular, the separation between the three studied strains progressively decreases with time. The three strains could still be identified despite the influence of culture time. Linear Discriminant Analysis (LDA) showed an overall accuracy of 88% and 71% in the training and test sets, respectively. These results indicate that the presence of microorganisms is detectable with respect to background, however, the difference between the strains changes with the incubation time.

## 1. Introduction

Fungal contamination of food and the environment may lead to the formation of products like mycotoxins and spores that can cause harmful effects to human health [1]. The fungi, *Aspergillus* has been the object of frequent studies. *Aspergillus* is a genus of filamentous fungi that contains around 250 species. *A. flavus*, *A. fumigatus*, and *A. niger* are the species most frequently involved in environmental contamination and associated with human diseases [2]. They are among the most abundant species in soil and for this reason they are also involved in disorders affecting plants and vegetable products [3,4]. In humans, *A. fumigatus* is an opportunistic pathogenic fungus that causes severe systemic infections and it is a major cause of fungal infections in immunocompromised patients [5]. It is classified in the second risk group according to its harmful properties (Italian Legislative Decree 81/2008). In addition to infectious diseases, *A. fumigatus* can induce allergic sensitization and symptomatic allergic lung disease [6] (invasive pulmonary aspergillosis, aspergilloma or different forms of hypersensitivity diseases), whose severity depends on the immunological status of the host.

With regard to the other species, both *A. flavus* and *A. niger* are of great interest in some occupational settings, especially in the agricultural and food sectors, as they are producers of mycotoxins, chemical toxic compounds that are produced via their secondary metabolism [7]. In suitable environmental conditions, *A. flavus* releases Aflatoxin B1 (AFB1), and it is the most toxic mycotoxin classified in Group 1 (carcinogenic to humans) by the International Agency for Research on Cancer (IARC). *A. niger* is a known producer of Ochratoxin A; it is a potent nephrotoxin and has teratogenic, immunosuppressive and carcinogenic properties (Group 2B, IARC, Lyon, France).

The standard methods used to identify microbial species include culturing and biochemical tests, immunological assays, examining the antigen—antibody interaction, and polymerase chain reaction (PCR) for DNA identification. Although reliable, specific, and sensitive, these approaches are also expensive, time-consuming and labor-intensive [8,9]. Biosensor technology is expected to eliminate the drawbacks associated with biochemical tests, even though most biosensors are oriented to the detection of toxins rather than to the presence of microorganisms [10].

Alternative methods for the detection of fungi consider specific metabolic products and the volatile metabolites, known as the volatilome. The analysis of volatile compounds (VOCs) is attracting attention in many different fields, including clinical diagnosis, environmental monitoring, and food contamination [11].

Microbial volatile organic compounds are metabolic products of fungi, and bacteria and are formed in both primary and secondary metabolism as side-products. An on-line VOCs database (http://bioinformatics.charite.de/mvoc/index.php?site=home) reports more than 1000 volatile organic compounds originating from microorganisms; over 300 of them are classified as fungal VOCs [12]. These VOCs include alcohols, aldehydes, hydrocarbons, acids, ethers, esters, ketones, terpenes, furans, sulfur and nitrogen-containing compounds. The profile of the produced compounds is strictly correlated with microbial species and strains, incubation time and growth conditions such as substrate, nutrients, pH, humidity and temperature [13].

Various analytical tools are suitable for detecting VOCs in different substrates. However, these techniques sometimes suffer from limitations that are typical of microbiological methods, that is, they often require sample pre-treatment, long-time analysis and complex data interpretation. Gas chromatography mass spectrometry is the typical standard for the analysis of gaseous compounds [14]. More advanced methods do not include the separation phase, and therefore, may have a fast analysis time [15]. These techniques have been applied to study *Aspergillus* volatilome in vitro and in real samples [16,17].

In general, these studies indicate that different microorganisms produce very similar volatile compounds, but the differences between them are found in the global pattern of VOCs rather than in single compounds. It is important to note that, even if some VOC may be associated with a given species, the actual pattern released by the microorganisms may be variable because various factors can alter the species-dependent pattern. Among these factors, the substrate and the growing conditions are of paramount importance. Because of such variabilities, the patterns released by replicated cultures are affected by dispersion. Thus, pattern recognition is necessary to correctly attribute a measured pattern to a species [18].

Patterns of VOCs can be obtained with analytical instruments, measuring the abundance of individual compounds. Arrays of cross-selective sensors (electronic noses) are an alternative method to access the same information. These are ensembles of partially selective sensors that encode high-dimension patterns of VOCs into a smaller-dimension pattern of sensors signals. The encoding can preserve the relevant information necessary for the identification of samples. In such cases, electronic noses ensure the rapid and simple analysis of complex matrices of VOCs, as demonstrated in different fields such as medical diagnosis [19,20,21], the environment [22] and foods [23].

Several studies have reported the use of electronic noses for the identification and differentiation of microorganisms in vitro [24,25], and in real samples such as cereal grain [26,27] and yoghurt [28].

In this paper, the headspace of three species of *Aspergillus* were measured by an array of porphyrins-functionalized quartz crystal microbalance (QMB) sensors to correlate volatile organic compound patterns with the specific fungal species and the growth time. The results show that sensors can detect the presence of contamination but the identification of species may vary with the cultivation time.

## 2. Materials and Methods

### 2.1. Samples

Three American Type Culture Collection (ATCC) fungal strains belonging to *Aspergillus* genus (*A. niger-9642*, *A. fumigatus-KM 8001*, *A. flavus-9643*, LCG Standard) were considered.

Fungi were previously inoculated in Sabouraud Liquid Broth (SLB) and then isolated on Potato Dextrose Agar (PDA) plates and incubated at 25 °C from 72 to 120 h in aerobic conditions.

In order to obtain cellular suspension at known concentrations, spores produced from different *Aspergillus* cultures were counted using a Burker counting chamber. Spore suspension from each *Aspergillus* species were plated onto replicated PDA petri dishes and incubated at 25 °C for 10 days. Petri dishes containing the same quantity of PDA substrate were preserved during the experiment, and the headspace of these dishes were used as reference.

Experiments were carried out at the Biological Agents Risk Laboratory of the Department of Occupational and Environmental Medicine, Epidemiology and Hygiene, INAIL (Monteporzio Catone, Rome, Italy).

### 2.2. The Electronic Nose

Fungi headspaces were measured using a gas sensor array designed and developed at the University of Rome Tor Vergata.

The array was made of eight quartz microbalance (QMB) sensors. In this device, mass changes (Δm) on the quartz surface result in a frequency shift (Δf) in the electrical output signal of the oscillator circuit. In the theory of small perturbations, Δf is linearly proportional to Δm [29]. The adopted QMBs were AT-cut quartz, with a fundamental frequency of 20 MHz, corresponding to a mass resolution of the order of a few nanograms/Hz.

The QMBs were functionalized by seven metal complexes (Mg, Co, Cu, Zn, Fe, Mn, Sn) and a free base (H_2_) of 5, 10, 15, 20-tetrakis-(4-butyloxyphenyl) porphyrin (TBPP) [30]. Porphyrin films were deposited on the quartz surface by spray casting.

Each QMB is connected to an oscillator circuit. The frequency of the output signals of the oscillators is sequentially measured using a reference clock provided by a temperature compensated quartz that achieves a frequency resolution equal to 0.1 Hz. The gas sensor array is complemented by temperature and relative humidity sensors. The sample is delivered through an embedded tubeless pneumatic system manifold. Input inlets are connected to a miniature diaphragm pump, a three-way valve, a proportional valve and a mass flow sensor. The sample flow was 50 sccm.

The instrument is connected and powered via a USB. In-house software in Matlab controls the instrument and the data acquisition.

Sensors were calibrated by measuring their response to a series of compounds that are representative of different chemical families, including cyclohexane, hexane, ethanol, xylene, styrene, hexanal, benzaldehyde, acetone, triethylamine, and acetic acid. The vapors of the volatile compounds were generated by bubbling a nitrogen gas stream into the liquid compound and diluting the vapor with nitrogen gas. The dilution rate was controlled by computer-driven mass-flow controllers (MKS). The concentration of the volatile compounds was calculated by Antoine’s law using the parameters listed in the NIST database (http://webbook.nist.gov/chemistry).

### 2.3. Headspace Analysis

Petri dishes containing *Aspergillus* culture were closed with an aluminum lid specifically designed for this experiment. The lid was shaped in order to seal the fungi culture from the surrounding environment to allow the formation of a headspace. About five minutes was necessary to establish a stable headspace composition. The volatile organic compounds released by *Aspergillus* were sampled and analyzed for one minute at a flow-rate of 50 sccm using the pump embedded in the gas sensor array. A filter with a porosity of 0.8 µm (Sartorius, Germany) was used during the analysis in order to avoid the migration of spores from the samples to the device. The reference samples required for a stable sensors baseline was obtained by flowing the laboratory air through a CaCl_2_ bed. Measurements were carried out under a biohazard hood.

**Figure 1:**
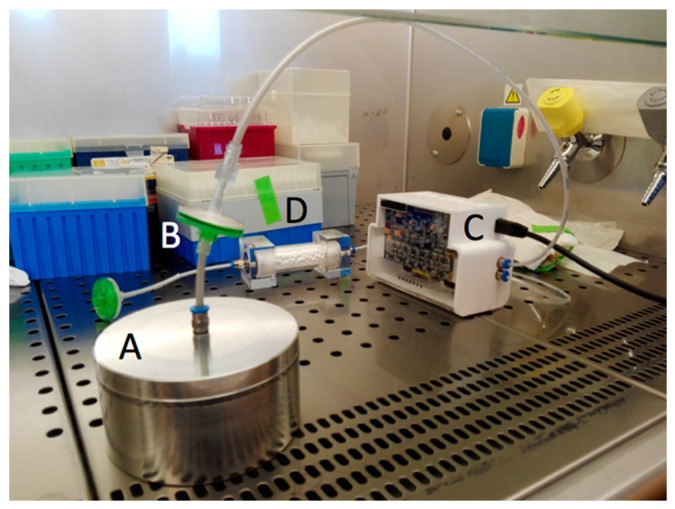
Picture showing the experimental system set-up under a biohazard hood. (**A**) Petri dish covered with customized lid suitable to ensure a stable headspace; (**B**) filter used to block spore migration from *Aspergillus* culture to sensors; (**C**) the electronic nose; (**D**) the CaCl_2_ trap.

Figure 1 shows the experimental set up. The cultures were analyzed 3, 5, and 10 days after the first inoculation.

### 2.4. Data Analysis

The sensor response consists of the variation in the resonant frequency measured at the beginning and at the end of the exposure to the sample. Each measurement results in an eight-dimensional vector, which is comprised of the responses of the individual sensors.

Data were analyzed using Principal Component Analysis (PCA) [31] to study the clustering of the sensors’ data with respect to the different species of fungi and to the temporal evolution of the VOCs. Linear Discriminant Analysis (LDA) [32] was used as a classifier to estimate the discrimination capabilities of the sensor array. The statistical significance of the sensor signals, and PCA scores were evaluated with the non-parametric Kruskal–Wallis rank sum test followed by Bonferroni correction in case of multiple comparisons.

All data analysis was performed in Matlab^®^ R2020a using Statistics and Machine Learning toolboxes.

## 3. Results and Discussion

Before we investigated the headspace of the microorganisms, the sensor array was characterized with respect to a set of generic volatile compounds. For this purpose, sensors were exposed to various concentrations of ten volatile compounds, which were chosen because they were most representative of different families. The concentration of the compounds was modulated by changing the dilution of the saturation pressure in a stream of nitrogen gas.

The sensors’ data were normalized by dividing the response of each sensor by the sum of the responses of all the sensors in the array [33]. The effects of different concentrations are largely removed in the normalized data and the VOCs can be identified without the confounding influence of variable concentrations [34].

**Figure 2:**
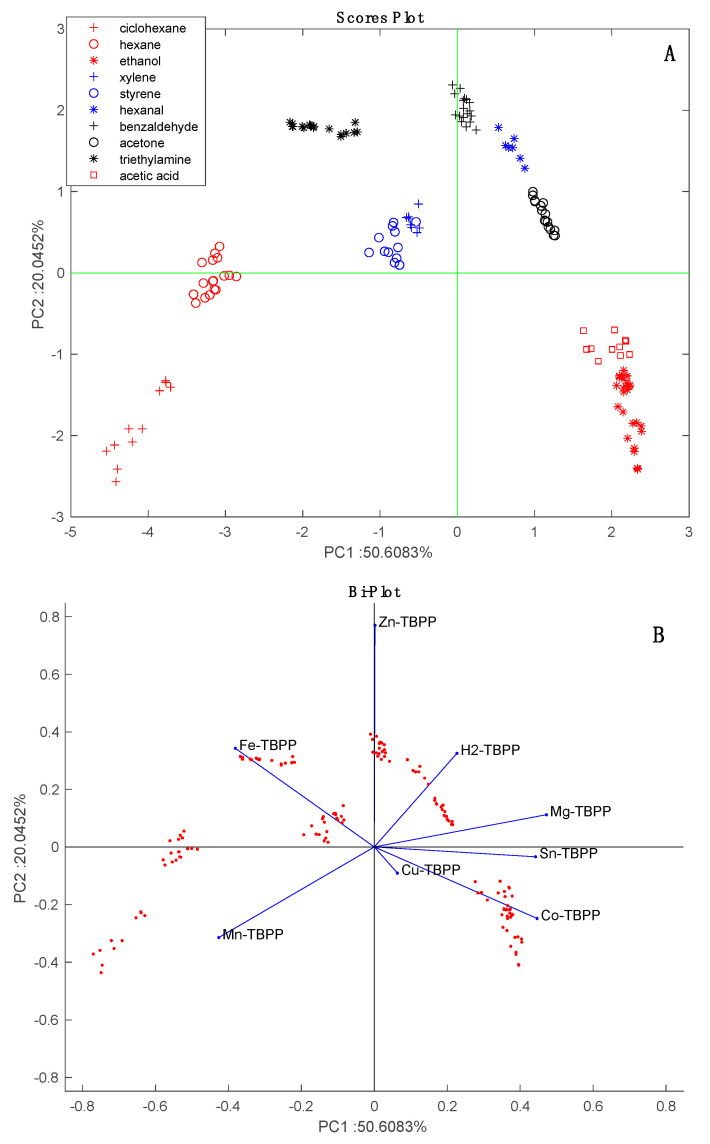
Principal Component Analysis (PCA) of characterization data. (**A**) Scores plot, (**B**) bi-plot which shows the loadings of the sensors.

Figure 2 shows the results of the PCA of the normalized characterization data. Except for the partial overlap of styrene and xylene, the data for each VOC form close clusters that are well separated from the others. The arrangement of VOCs in the scores plot is consistent with the chemical families. The sensors are uniformly distributed in the bi-plot. For instance, Mn-TBPP and Fe-TBPP make a large contribution with respect to alkanes (cyclohexane and hexane), while Co-TBPP is dominant with respect to polar compounds (ethanol and acetic acid).

The analysis of *Aspergillus* spp. headspace was carried out in replicated cultures in order to examine the biological variability in independent cultures.

**Figure 3:**
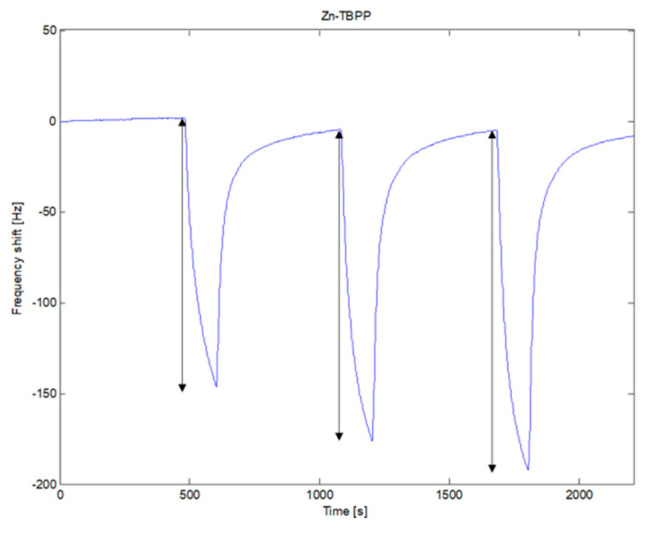
Signal recorded by the Zn-TBPP sensor. It shows the sensor behavior for the sequence of exposures to the headspace of *A. niger*, *A. flavus* and *A. fumigatus* in order. Arrows indicates the frequency shift considered as the sensor response and used in the data analysis. The response time is 135 s.

Figure 3 shows the sequence of the signals recorded by one of the sensors (Zn-TBPP) during three consecutive measurements of the headspace of cultures of *A. niger*, *A. flavus* and *A. fumigatus*. After each exposure to culture VOCs, the signal returns to its original baseline. The reversibility of the sensors is not absolute, however, such a limited hysteresis does not affect the result of the analysis.

The absorption of VOCs increases the mass on the QMB, then the frequency shift is negative. However, it is usual to consider the absolute value of the difference of the frequency before and at the end of the exposure as the sensor response. This value was used for all the following analysis.

**Figure 4:**
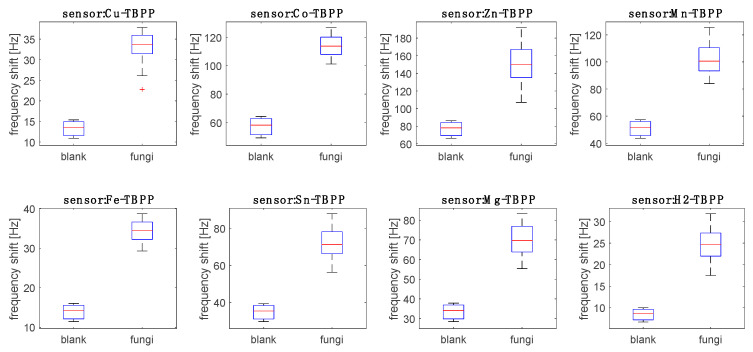
Distribution of the sensor responses to uncontaminated culture media and media inoculated with fungi. Each group contains data obtained at days 3, 5, and 10 after the first inoculation. The distribution of the responses is represented by a box-plot calculated with the Matlab embedded function. In each box, the central mark is the median, the edges are the 25th and 75th percentiles, the whiskers extend to the most extreme data points not considered outliers. Outliers, labeled with a cross, are plotted individually.

Figure 4 shows the response of the sensors to the headspace of fungi compared to the response to blank samples (PDA) measured after 3, 5 and 10 days. Blank PDA dishes were not inoculated with *Aspergillus*, however, they were left exposed to the laboratory air. Environmental contamination was not tested but it could not be excluded.

All sensors’ signals show a large and statistically meaningful difference between fungi and PDA. Thus, the VOCs released by blank culture media during the experiment did not have an effect on the sensors’ response to fungi.

The sensors’ data for the *Aspergillus* headspace were sorted into nine groups corresponding to the three strains and to the three inoculation times (days 3, 5, and 10). Figure 4 shows the distribution of the responses of the sensors with respect to the nine groups.

**Figure 5:**
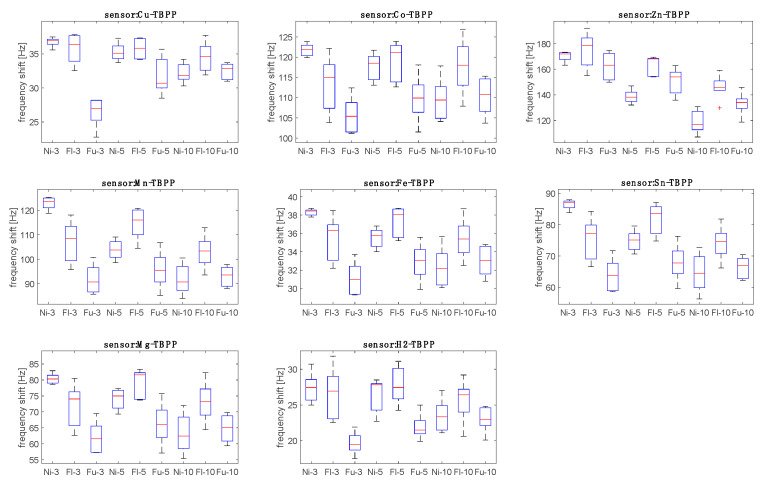
The distributions of the responses of sensors in the nine groups are represented by box-plots. Groups are labeled with the initials of the strain (Ni, Flu, and Fu) and a number (3, 5, 10) indicating the days since inoculation. For a description of the box-plot details, see the caption of Figure 4.

Figure 5 shows that both strain and time of culture influence the response of the sensors. The sensors demonstrate similar behavior, and in this regard, it is important to note that most of the correlation between the sensors’ data is due to the abundance of VOCs in the headspace. The relative response to the three strains changes with the time since inoculation.

This suggests that the real-time evolution of the volatilome composition is nonlinear. Previous studies suggest that the relationship between the abundance of volatile compounds and the time from inoculation shows particular trends for each compound [35]. For instance, in *Aspergillus fumigatus*, the abundance of some ketones (2-pentanone, 2-heptanone, 2-octanone and 2-nonanone) strongly decreased after three days while the changes in alcohol content was observed after seven days. Different behaviors were observed for alcohols. For example, from day 10 to day 14, the abundance of 3-Methyl-1-butanol decreased in *Aspergillus fumigatus* but increased in *Aspergillus niger* [36].

**Figure 6:**
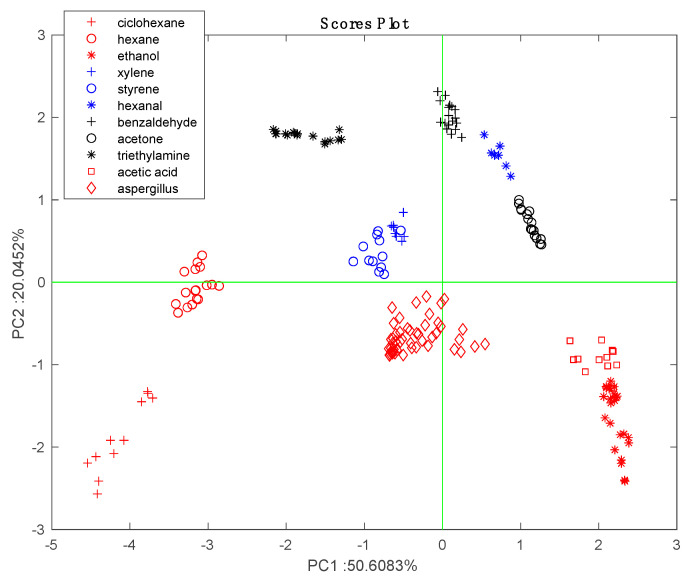
Sensors’ data related to *Aspergillus* headspaces were normalized and projected onto the scores plot of the PCA calculated with the characterization data and shown in Figure 2. Microorganisms’ data form a close cluster between aromatics and polar compounds.

Before further analyzing the data in order to determine the differences between strains it is interesting to compare the responses to *Aspergillus* species with responses to the pure VOCs. The data regarding the headspace of the microorganism headspace was projected onto the scores plot shown in Figure 2. Figure 6 shows that with respect to pure VOCs, *Aspergillus* data form a compact cluster lying between alcohols, aromatics, and alkanes. This result is consistent with the fact that alcohols (2-methyl-1-propanol and 2-ethyl-hexanol), ketones (2,4,pentanedione and 2-heptanone) and aromatics (3-methylfurane) are among the most frequently identified components of *Aspergillus* volatilome [16].

A better appraisal of the sensors’ data can be achieved by considering the multivariate analysis of the sensor responses. Hence, PCA and LDA were applied to the data.

PCA is an unsupervised algorithm that is useful to study the correlations between data and the correlations between sensors. On the other hand, LDA, being supervised, provides an estimate of the ability of the sensor array to identify the various groups.

Before calculating the PCA, the data was normalized by dividing the sensor signals by the sum of signals of all sensors.

**Figure 7:**
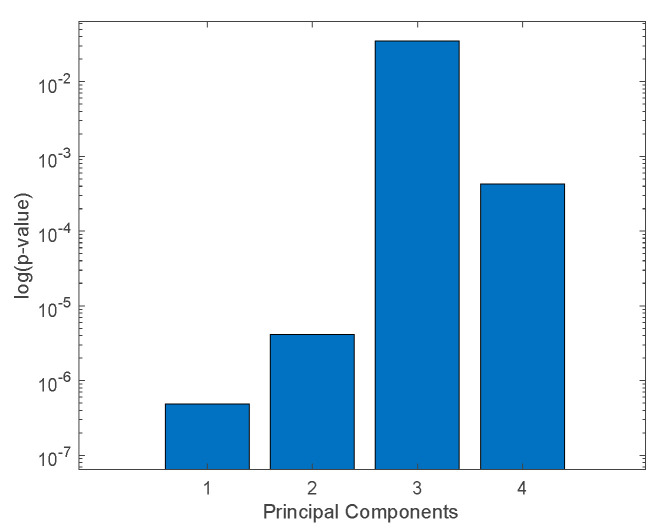
Plot of the logarithm of the probability of null hypothesis (*p*-value) returned by a non-parametric Kruskal–Wallis rank sum. The *p*-value is related to the largest separation between at least two of the nine groups.

Analysis of variance (non-parametric Kruskal–Wallis rank sum) was applied to the principal components in order to select those that achieved the largest separation of the *Aspergillus* species. Figure 7 shows the corresponding *p*-value calculated for the first four principal components that explain more than 1% of the total variance. The first two principal components (which explain 81.5% and 14.5% of the total variance, respectively) show the largest separation among the classes.

**Figure 8:**
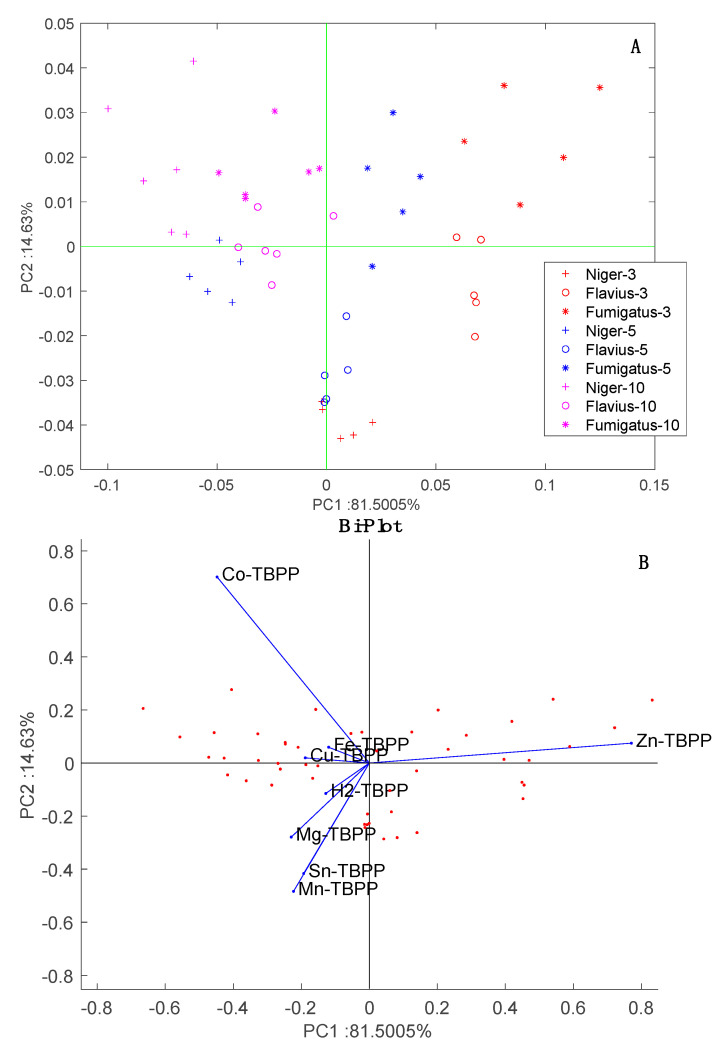
PCA of *Aspergillus* headspace data. (**A**) scores plot, (**B**) bi-plot where the loadings of sensors are indicated. In Figure 8A, *Aspergillus* species are marked with the same symbol whose colors identifies the time after incubation.

The scores in the plane of PC1 and PC2 are plotted in Figure 8. This plot shows the two influences on the data: strain and incubation time. The effect of the incubation time is observed along PC1 while the separation between the strains is observed along PC2. Since the variance explained by PC1 is more than five times larger than that explained by PC2, we can conclude that the largest variability in the data is due to the change in volatilome occurring as the cultures grow. It is important to note that time has no influence on the data from the blank samples, thus, the effect cannot be merely due to sensors drift.

The loading of the sensors to the PCA scores plot are shown in Figure 8B. With respect to PC1, all sensors are oriented towards the increment of time except Zn-TBPP. With respect to PC2, Co-TBPP singularly points to *A. fumigatus* samples. In Figure 2B, these sensors contribute most to aldehydes and amines (Zn-TBPP) and polar compounds (Co-TBPP). Thus, based on his behavior we can surmise a prevalence of polar compounds in *A. fumigatus* and a decrease in aldehydes along the incubation time. Further investigation is necessary to confirm this conjecture.

Although, incubation time is the dominant source of variability in the data, we also wanted to investigate if the data contain also enough information to identify the strains regardless of their incubation time. Therefore, LDA was used to calculate a classifier for the recognition of three strains, *A. niger*, *A. flavus*, and *A. fumigatus*.

**Figure 9:**
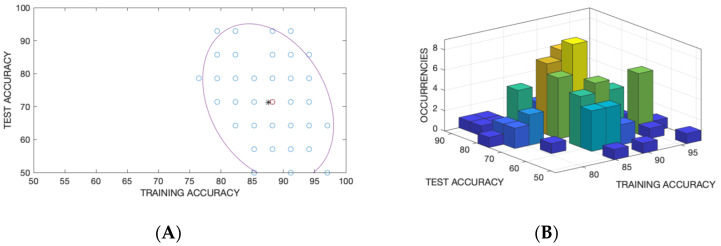
(**A**) Plot of accuracy of the Linear Discriminant Analysis (LDA) classifier for 100 random partitions of the data in training and test sets. (**B**) Frequency of occurrence of the multivariate variable made by the accuracy in training and test sets. The ellipse in Figure 9A shows the covariance matrix of the bi-variate normal distribution. The mean value, corresponding to the center of the covariance matrix, was chosen to represent the results.

Due to the limited sample size, the classification performance is strongly dependent on how the data are split in the training and test sets. To avoid misinterpretation of the data, the whole dataset was randomly split into training and test sets and the LDA was calculated for each split. Figure 9 shows the accuracy in the training and test sets for each random split. The accuracies distribution is well fitted by a bivariate gaussian. In Figure 9A, the mean and the ellipsoid associated to the covariance matrix are shown while in Figure 9B the occurrence of a couple of accuracies in training and test are shown.

Table 1 shows the confusion matrix for the mean of the distribution of accuracy in the training and test sets. In total, about 88.2% of the samples were correctly identified in training and 71.4% in test.

Thus, even if the variability due to the different strains is residual with respect to the incubation time, it enables the identification of the species of *Aspergillus*.

## 4. Conclusions

This paper reports the application of a gas sensor array to in vitro discrimination of *A. niger*, *A. fumigatus* and *A. flavus* species, which are often involved in environmental and food contamination. Previous studies, carried out with analytical instrumentation, have shown that each species is characterized by a unique pattern of VOCs. However, the abundance of each VOC and its evolution along the cultivation time is variable and it depends on the species, as well as the culture media.

Sensors detect the presence of the fungi with respect to the blank sample, but the difference between the species depends on the cultivation time. This finding is corroborated by previous investigations that show that the abundance of each VOC has a proper rate of evolution with the cultivation time. It is particularly interesting that very similar compounds, for which sensors are expected to have the same sensitivity, may decrease in *Aspergillus fumigatus* but increase in *Aspergillus niger* [33]. This behavior suggests the possibility of detecting microorganisms independently from the growth conditions (time and culture media). Our data show that the processes occurring along the incubation time contributes significantly to the variance in the data; however, even though it is not predominant, the information related to the strain is captured by the sensors and can be used to identify the strains independently of the incubation conditions.

In this study the performance of the sensors was not considered to be optimal for practical routine applications. On the other hand, the composition of the sensor array was not optimized for this purpose. A detailed investigation of the chemistry of *Aspergillus* genus volatilome could be used to make a careful choice of sensitive materials to design electronic noses that could be used for rapid and non-invasive detection of fungal contamination.

Such studies will require a wider experimental design that includes growth in different media and incubation parameters (e.g., temperature and humidity). Furthermore, although the chosen species are among the most interesting in the *Aspergillus* genus, an extension of the study to other members of the genus should be considered for a thorough appraisal of the ability of electronic noses to identify similar microorganisms.

## Figures and Tables

**Table 1:** Confusion matrix of the LDA model in training and test. Correct identifications are emphasized with bold font.

	***Training***		
	***Asp. Niger***	***Asp. Flavius***	***Asp. Fumigatus***
*Asp. Niger*	**11**	1	1
*Asp. Flavius*	1	**8**	0
*Asp Fumigatus*	0	1	**11**
	***Test***		
	***Asp. Niger***	***Asp. Flavius***	***Asp. Fumigatus***
*Asp. Niger*	**4**	1	0
*Asp. Flavius*	0	**2**	0
*Asp Fumigatus*	0	3	**4**
